# Women’s experience and satisfaction with midwife-led maternity care: a cross-sectional survey in China

**DOI:** 10.1186/s12884-021-03638-3

**Published:** 2021-02-19

**Authors:** Ying Liu, Tengteng Li, Nafei Guo, Hui Jiang, Yuehong Li, Chenying Xu, Xiao Yao

**Affiliations:** 1 Nursing Department, Shanghai First Maternity and Infant Hospital, Tongji University School of Medicine, No.2699, West Gaoke Road, Pudong New Area, Shanghai, 201204 China; 2 Delivery Room, Shanghai First Maternity and Infant Hospital, Tongji University School of Medicine, No.2699, West Gaoke Road, Pudong New Area, Shanghai, 201204 China

**Keywords:** Maternity care, Midwife, Pregnancy, Vaginal delivery, China

## Abstract

**Background:**

Low risk pregnancy ending in a vaginal birth is best served and guided by a midwife. Utilizing a midwife in such cases offers many emotional and economic advantages and does not increase the risks for mother or neonate. However, women’s experience and satisfaction of midwife-led maternity care is rarely reported in China. The primary objective of this study is to describe the experience of Chinese women receiving midwife-led maternity care, and to report their satisfaction level of the experience.

**Methods:**

The study is a cross-sectional survey of 4192 women who had natural birth from March–June 2019 in a maternity care center, Shanghai, China. We used a self-administered questionnaire addressing items related to women’s experience during childbirth, as well as their satisfaction with midwife-led maternity care. We also included demographic and perinatal characteristics of each participant. Descriptive statistics and correlations analysis between groups of different experience and satisfaction were used.

**Results:**

In this sample, 87.7% of women had a Doula and a family member present during childbirth. Epidural anesthesia was used in 75.6% and episiotomy was needed in 23.2%. Free positioning during the first stage of labor and free positioning during the second stage of labor and delivery were adopted in 84.3 and 67.9% of the cases, respectively. Moderate to severe perineal pain and moderate to severe perineal edema were reported in 43.1 and 12.2% of the participants, respectively. High satisfaction level was found when there was midwife-led prenatal counseling and presence of Doula and family member, Lamaze breathing techniques, warm perineal compresses, epidural anesthesia, free positioning during the first stage of labor, and midwifes’ postpartum guidance. Negative satisfaction was seen with perineal pain and edema.

**Conclusion:**

Women in this survey generally had high satisfaction with midwife-led maternity care. This satisfaction is probably felt because of the prenatal counseling by the midwife and allowing a Doula and a family member in the room during childbirth. Other intangible factors to improve the satisfaction level were Lamaze breathing techniques, warm perineal compresses, epidural anesthesia, free positioning during first stage of labor, and early skin to skin contact.

**Supplementary Information:**

The online version contains supplementary material available at 10.1186/s12884-021-03638-3.

## Background

Natural birth guided by a midwife is the optimal service for low-risk pregnancy without increasing the risks for mothers and neonates [1]. Two studies in Shanghai found women’s choices of delivery, are influenced by complex internal and external factors [2, 3]. Vaginal delivery is motivated by factors such as fast recovery, immediate breastfeeding, and powerful bonding [4]. In addition, women delivering vaginally reported greater fulfillment and less distress than those delivering by cesarean section (C-S) even many years post-delivery [5]. Wang reported that labor pain and lack of social support during childbirth were the major reasons for women to request a C-S [6]. These factors may explain the high C-S rates in China which remains 41.1–45.6% (nationwide from 2012 to 2016) [7]. Women tend to view the C-S as a way to avoid pain, when in fact a C-S is not devoid of pain and carries many adverse maternal and neonatal outcomes [8, 9]. All across the World, there is a growing interest for midwife or a team of midwives leading the planning, organization, and delivery of care, with some consultation from obstetricians [10–12]. This midwife-led team compared to obstetrician-led maternity care was associated with lower maternal and neonatal mortality, lower C-S rate, lower and better postpartum wellbeing [10, 12–14]. In the scope of midwifery, according to the framework for quality maternal and newborn care, effective practice for childbearing women and infants includes education, information, health promotion and public health; assessment, screening and care planning; promotion of normal process and prevention of complications; first-line management of complication and so on [12].

Maternity care in China is predominantly hospital-based and obstetrician-led [15]. With the Chinese modernization campaign in the 1980s, a medical model had been widely used and led to medicalization and hospitalization of childbirth throughout China [16]. By 2018, hospital delivery rate approaches 100% especially in large cities such as Shanghai and Beijing. As the largest developing and the largest population country, China bears a substantial burden of maternity care and faces a serious shortage of obstetricians, obstetric nurses, and midwives. This fact prompted the National Health Commission of the People’s Republic of China to release the Maternal and Child Safety Action Plan (2018–2020) in May 2018. This action plan aimed at advocating women-centered hospital delivery services and establishing a safe and comfortable delivery environment for women.

In China, there is no nationally recognized registration system of midwives [17]. Midwife only certified by some local health bureau. Midwifery is a branch of nursing, and all midwives have to be a registered nurse with nursing licenses [15, 18]. Midwives are mainly responsible for providing care during labor collaborating with other health professionals and being supervised by obstetricians [18, 19]. To be a midwife, first, they have to finish their education in midwifery for 3–4 years in college or university, some may have a nursing education background [17, 20]. Then they should pass the nurse qualification examination, and achieve all the requirements to become a registered nurse [20]. Besides this, to get a midwife certification from the local health bureau, they must have at least one year of working experience in the labor room, and receive specific training and pass the qualification test. With certificates, they can be employed and provide care in hospitals, and their certificates should be recertified every two years.

Over the past decade there had been substantial reform in providing maternal and child health care in China [21]. Due to lacking bachelor degree education in midwifery, and being a sub-branch of the nursing profession, the development of the midwifery profession in China is limited [15]. Still, midwives are incontrovertible important birth-care providers in China and gradually starts to play a leading role in providing intrapartum care for women with normal labor in the hospital delivery room especially in obstetrics and gynecology hospital [13, 16, 22]. Some midwifery initiatives were launched to revive the importance of the profession. In 2007, a midwife-led normal birth unit was developed in Hangzhou, which now acted as an example of midwife-led maternity care in China [23]. The China Maternal and Child Health Association also started a series of measures to promote vaginal delivery and midwifery care [7]. By 2014, eight regional training centers were providing midwifery training. Tertiary hospitals now have a cadre of midwives to manage labor and delivery [7]. Now women with 37–41 weeks of gestation, a singleton pregnancy, cephalic presentation, longitudinal lie, normal cardiotocograph are encouraged to try a vaginal birth in the hospital labor ward with the help of midwives. A midwife can also provide prenatal counseling and birth planning in the midwife clinic and offer childbirth education and psychological support to women in a postpartum obstetric ward in China [15].

As the one-child policy fades and the new universal two-child policy unfolded in China [24], more Chinese families can expect a second child. Clinicians and women are choosing natural vaginal delivery for the first pregnancy to avoid a repeated C-S and its associated risks [7]. This new trend towards encouraging and supporting natural birth elevates the importance of midwives in assisting vaginal delivery. The shift in policy and concerns for the risk of C-S [25], coupled with new guidelines to promote natural birth are producing tangible results in decreasing C-S rates in cities like Shanghai [26]. We need to look at the effect of all these changes on the quality of care and patients’ safety, as well as assess the overall level of patients’ satisfaction resulting from this shift in health care paradigms. The aim of this study was: (1) to describe the women’s experience and satisfaction of midwife-led maternity care in Shanghai, China. Experience items directly and indirectly related to midwifery care practice were included. Such as midwife-led prenatal counseling, doula and family member at delivery, midwives’ postpartum guidance, women’s perineum pain 2 h after delivery. (2) to explore the relationship between women’s experience and satisfaction and to investigate the predictiors of women’s satisfaction.

## Methods

### Study design, setting, and participants

This was a cross-sectional, descriptive survey conducted among women receiving midwife-led maternity care in Shanghai, China. A self-developed questionnaire was used to investigate women’s experience and satisfaction with childbirth. The impact of women’s experience on their satisfaction with midwife-led maternity care was examined, and demographic and perinatal variables related to women’s satisfaction were included as confounding factors.

The study was conducted in Shanghai First Maternity and Infant hospital. It is one of the 3-A-Class Obstetrics and Gynecology Specialized Hospitals in Shanghai and it’s been a baby-friendly Hospital since 1992. The hospital is a 749-bed facility, with 400 beds for obstetrics wards, 225 beds for gynecology wards, 14 beds for labor room, and 110 beds for neonate ward. It is one of China’s largest providers of maternity care with about 30,000 deliveries per year. The C-S rate was about 41%. Over the study period (4 months), there were about 5191 vaginal births in this hospital, and 4989 (96.1%) of them received midwife-led care, only 202 (3.9%) of them received doctor-led care. For women who had C-S, they all received doctor-led care. The general hospital stay was 36 h after vaginal birth and 48–60 h after C-S.

In this setting, according to Renfrew framework of maternal and newborn care [12], the health policy in China [17, 20], and the local and hospital regulations [13], the midwife-led care is provided by midwives who are hired and work in the hospital providing intrapartum care in the labor room and part of antenatal care and postnatal care to women with low-risk pregnancy and women planned to have a natural birth.

Midwives usually provide antenatal care through midwife clinics. For all pregnant women in China, antenatal care is usually provided by obstetricians and midwives’ antenatal clinical services are only available in some teaching hospitals in developed cities. In this setting, the midwife clinic is available as part of midwife-led maternal care. For women who plans to have a natural birth, they are advised to go to the midwife clinic. In the clinic, they could get a one-to-one individual consult by a senior midwife, and personalized antenatal care would be given, including health education on exercise, nutrition, weight management, birth plan, birth skill, and mother-fetal monitory. Intrapartum care usually starts when the women are transferred into the labor room from the Emergency Room or obstetrics wards (usually when cervical dilation is 2–3 cm), and ends when the women are transferred to the obstetrics ward (usually 2–3 h after delivery). Intrapartum care includes educating the women, assessing labor progress, providing continuous emotional support during labor, encouraging mobility in labor, offering pain relief measures, delivering the newborn, performing an episiotomy and repairing perineal if necessary, and facilitating skin-to-skin contact. The postnatal care includes immediate postpartum care in the labor room, postpartum visit in the obstetric wards, and postpartum recovery consulting through the midwife clinic. To promote continuity of care, the postpartum visit is usually given by the midwife who takes main responsibility during intrapartum care, and the visiting time is the next morning after delivery before the women discharge from the hospital. Assessing the postpartum recovery process, health education, and facilitating breastfeeding are provided.

All women who have received midwife-led care and met the inclusion criteria were consecutively enrolled. The study inclusion criteria were (1) Age greater than 18 years; (2) Singleton birth; (3) Gestational age greater than 32 weeks; (4) Apgar score greater than 7 at 1 min; (5) Competency in Mandarin; (6) Ablity to access a mobile phone and complete an electronic questionnaire. All women who have received midwife-led care and met the inclusion criteria were consecutive enrolled, and only women who refused to participate in the study were excluded.

There were 4832 eligible women in the study period who were approached and 4216 of them agree to participate. Then 4145 unique questionnaires were received (71 duplicate cases were eliminated), and after a careful examination of all the data, 24 cases with missing data were eliminated. Finally, a total of 4192 participants remained (86.8% response), corresponding to 80.8% of all vaginal delivery population during the study period.

### Data collection

The study was conducted over a 4 months period (March 2019–June 2019). The data collection was conducted in the labor room. The research staff approached eligible women, who were 2–3 h postpartum after delivery, have finished the routine postpartum assessment and education provided by midwives, ready, and waiting to be transferred to the obstetric wards. The research staff informed them the purpose and procedure of the study and women were reassured that refusal to participate will not affect their care. After obtaining informed consent, the research staff instructed the participants to fill in the questionnaire on their mobile phones through the electronic questionnaire connected to the WeChat app. Adequate time was allowed for participants to ask questions and rest if they have any problems with the questionnaire. And if bad experience was reported by the women, the next morning after delivery, the midwife would go to the obstetrics ward and give a routine postpartum visit. During the visit, the bad experience would be discussed and women’s suggestions or advices for improving midwife-led care were collected.

### Instrument

Data were collected using a self-administered questionnaire that had three sections: demographics, experience, and satisfaction section (see supplementary file). The questionnaire was developed from literature reviews, experts’ opinions, and current practice of midwifery care in the hospital to address the study objectives.

(1) Demographic section: Demographic and perinatal factors that may affect women’ s satisfaction with childbirth were included, such as women’s age, household registration location, parity, gestational age, sex of neonate, weight of neonate (Table 1). (2) Experiences section: A 13-item questionnaire was developed specially for this study (Table 2). Experience directly related to midwifery care practice, such as midwife-led prenatal counseling (antenatal care), presence of doula and family member at delivery (intrapartum care), and midwives’ postpartum guidance (postnatal care), and experience indirectly related to midwifery care practice such as perineum pain 2 h after delivery. Ten Yes or No Experience items included: midwife-led prenatal counseling, presence of doula and family member at delivery, Lamaze breathing techniques, warm perineal compresses, epidural anesthesia, free positioning during the first and second stage of labor, episiotomy, early skin to skin contact, and midwifes’ postpartum guidance. And three other experience items such as perineal laceration, perineal pain, and perineal edema were rated from 1 to 4 (1 = absence and 4 = severe degree).

**Table 1:** Relationships of the satisfaction score with demographic and perinatal characteristics of the participants (*N* = 4192)

Variables	***n***(%)	Satisfaction Mean ± SD	t/F value	***p*** value
**Age**			1.182	0.237
18–34 y	3721 (88.8)	8.957 ± 0.77		
≥ 35 y	471 (11.2)	8.912 ± 0.77		
**Household registration location**			4.457	**< 0.001**
Shanghai	1303 (31.1)	9.03 ± 0.72		
Non-Shanghai	2889 (68.9)	8.92 ± 0.79		
**Parity**			9.946	**< 0.001**
Primipara	3106 (74.1)	9.02 ± 0.73		
Multipara	1086 (25.9)	8.75 ± 0.83		
**Gestational age**			−2.841	**0.005**
Preterm (196–258 days)	143 (3.4)	8.77 ± 0.93		
Full term (259–293 days)	4049 (96.6)	8.96 ± 0.76		
**Sex of neonate**			2.984	**0.003**
Boy	2192 (52.3)	8.99 ± 0.75		
Girl	2000 (47.7)	8.92 ± 0.79		
**Weight of neonate**			3.882	**0.021**
Low birth weight (1500-2499 g)	83 (2.0)	8.72 ± 0.81		
Nomal birth weight (2500-3999 g)	3954 (94.3)	8.96 ± 0.77		
Fetal macrosomia (≥4000 g)	155 (3.7)	8.94 ± 0.69

**Table 2:** Relationships of the satisfaction score with women’s experience during childbirth of the participants (*N* = 4192)

Experience item	***n***(%)	Satisfaction Mean ± SD	t/F value	***p*** value
**Midwife-led prenatal counseling**			−25.496	**< 0.001**
No	1945 (46.4)	8.65 ± 0.75		
Yes	2247 (53.6)	9.21 ± 0.69		
**Presence of doula and family member at delivery**			−15.452	**< 0.001**
No	515 (12.3)	8.47 ± 0.89		
Yes	3677 (87.7)	9.02 ± 0.73		
**Lamaze breathing techniques**			−22.597	**< 0.001**
No	2468 (58.9)	8.74 ± 0.76		
Yes	1724 (41.1)	9.26 ± 0.67		
**Warm perineal compresses with red-bean bags**			−25.145	**< 0.001**
No	2929 (69.9)	8.77 ± 0.76		
Yes	1263 (30.1)	9.38 ± 0.60		
**Epidural anesthesia**			−22.560	**< 0.001**
No	1024 (24.4)	8.51 ± 0.82		
Yes	3168 (75.6)	9.10 ± 0.70		
**Free position during the first stage of labor**			−8.496	**< 0.001**
No	659 (15.7)	8.72 ± 0.86		
Yes	3533 (84.3)	9.00 ± 0.74		
**Free position during the second stage of labor**			−1.092	0.275
No	1345 (32.1)	8.93 ± 0.75		
Yes	2847 (67.9)	8.96 ± 0.78		
**Episiotomy**			−2.313	**0.021**
No	3221 (76.8)	8.94 ± 0.78		
Yes	971 (23.2)	9.00 ± 0.73		
**Laceration of perineum**			3.530	**0.014**
No	1053 (25.1)	8.96 ± 0.75		
l^st^ degree	1596 (38.1)	8.91 ± 0.79		
2nd degree	1541 (36.8)	8.99 ± 0.76		
3rd degree	2 (0.0)	9.40 ± 0.42		
**Early skin to skin contact**			−1.319	0.187
No	2476 (59.1)	8.92 ± 0.77		
Yes	1716 (40.9)	9.01 ± 0.65		
**Midwives’ postpartum guidance**			−12.984	**< 0.001**
No	92 (2.2)	7.94 ± 1.11		
Yes	4100 (97.8)	8.97 ± 0.74		
**Perineum pain two hours after delivery**			4.105	**0.006**
Almost no pain	606 (14.5)	9.05 ± 0.76		
Mild pain	1778 (42.4)	8.95 ± 0.75		
Moderate pain	1112 (26.5)	8.91 ± 0.81		
Severe pain	696 (16.6)	8.93 ± 0.75		
**Perineum edema two hours after delivery**			5.314	**0.001**
Almost no edema	1831 (43.7)	9.00 ± 0.71		
Mild edema	1850 (44.1)	8.92 ± 0.81		
Moderate edema	450 (10.7)	8.88 ± 0.82		
Severe edema	61 (1.5)	8.94 ± 0.71		

(3) Satisfaction section: Women’s satisfaction with the childbirth experience was rated 1 to 10 (1 = not satisfied, 10 = completely satisfied).

### Ethical considerations

Ethical approval was obtained from the hospital Institutional Review Board. Furthermore informed consent was obtained from every participant. Participants were assured that the information collected in this study was confidential and anonymous, and their participation was totally voluntary.

### Data analysis

Data were analyzed using the Statistical Package for Social Science (SPSS) 22.0 version for Windows. Demographic characteristics, labor experience, and satisfaction are reported using descriptive statistics. Shapiro-Wilk normality test was used to verify the normality of variables [27]. Group differences of satisfaction were tested by t-test or one-way ANOVA. Multiple linear regression analysis was performed to explore the predictors of satisfaction scores. Women’s experience that related to satisfaction in univariate analysis (*p* <  0.05) were brought into the multiple regression analysis with the stepwise procedure (entry 0.05, removal 0.10), and demographic variable added as control variables. The alpha level of significance was set at 0.05.

## Results

### Characteristics of the participants and satisfaction (Table 1)

Table 1 describes the demographic and perinatal characteristics of the participants. Age ranged from 18 to 45 years (average 29.8 years). The majority of women were primipara (74.1%). The gestational age of neonates ranged from 210 to 290 days (mean 276.4 ± 8.5 days). The average score of women’s satisfaction with midwife-led maternity care was 9.0 ± 0.8. The univariable analysis showed that women’s household registration location, parity, gestational age, sex of neonate, weight of neonate were factors significantly related to women’s satisfaction with childbirth.

### Women’s experience (Table 2)

In this study, as a component of antenatal care, only 53.6% of women received midwife-led prenatal counseling. With regards to intrapartum care, 87.7% of deliveries had a Doula or a family member and only 23.2% had episiotomy. Labor pain was controlled with epidural anesthesia in 75.6%. Hot perineal compresses with red-bean bags were used in 30.1% while Lamaze breathing technique was utilized by 40.1%. 97.7% of women reported that they got postpartum guidance from midwives. The univariable analysis showed that midwife-led prenatal counseling, presence of doula and family member at delivery, Lamaze breathing techniques, warm perineal compresses with red-bean bags, epidural anesthesia, free position during the first stage of labor, episiotomy, laceration of perineum, midwives’ postpartum guidance, perineum pain two hours after delivery, and perineum edema two hours after delivery were significantly related to women’s satisfaction with childbirth.

### Women’s experience associated with satisfaction (Table 3)

Multiple linear regression analysis was conducted to investigate the predictiors of women’s satisfaction. As shown in Table 3, experience with midwife-led prenatal counseling, Doula and family member presence, Lamaze breathing techniques, warm perineal compress, epidural anesthesia, free positioning during the first stage of labor, and midwives’ postpartum guidance were positively associated with women’s satisfaction.

**Table 3:** Multiple linear regression analysis of factors associated with women’s satisfaction

	B	SE	β	t	P value†
(Constant)	7.088	0.157		45.064	< 0.001
Household registration location (Ref:Shanghai)					
Non-Shanghai	−0.052	0.02	−0.031	−2.541	0.011
Sex of neonate (Ref: boy)					
girl	−0.043	0.019	−0.028	−2.311	0.021
Midwife-led prenatal counseling (Ref: no)					
yes	0.356	0.02	0.231	17.954	< 0.001
Presence of doula and family member at delivery (Ref: no)					
yes	0.292	0.03	0.125	9.681	< 0.001
Lamaze breathing techniques (Ref: no)					
yes	0.319	0.02	0.204	16.048	< 0.001
Warm perineal compresses with red-bean bags (Ref: no)					
yes	0.445	0.021	0.265	21.457	< 0.001
Epidural anesthesia (Ref: no)					
yes	0.413	0.025	0.23	16.784	< 0.001
Free position during the first stage of labor (Ref: no)					
yes	0.077	0.027	0.036	2.863	0.004
Midwives’ postpartum guidance (Ref: no)					
yes	0.787	0.064	0.15	12.316	< 0.001

† Parity, gestational age, weight of neonate, episiotomy, laceration of perineum, perineum pain 2 h after delivery, perineum edema 2 h after delivery did not statistically affect satisfaction (*P* > 0.05)

## Discussion

In this study, women’s experience and satisfaction with midwife-led maternity care were investigated, as well as their correlation. Firstly, We will discuss the experiences items that significantly related to women’s satisfaction with childbirth. Then the experiences items, that did not show significant relationships with satisfaction but actually were very important indicators to reflect the quality of care, will be discussed.

Midwife-led interventions are associated with efficient use of resources and have a positive impact on maternal health outcomes [12]. Midwives, trained with ICM global standards could play an important role in maternal and newborn care [12]. A recent study highlights the substantial potential of midwives (trained according to ICM standards) as a single occupation group to contribute to reducing maternal and neonatal mortality and stillbirths if they are a part of a multidisciplinary team operating within an enabling environment [14]. In this study, in one of the high-level Maternity and Infant hospitals in Shanghai, midwives are registered nurses with nursing licenses and get midwifery certification from the Shanghai health bureau. They are competent to provide intrapartum care in the labor room and a part of antenatal care and postnatal care to women with low-risk pregnancies and planned to have a natural birth. And we found that midwifery practice, such as prenatal counseling, labor companion, pain relief & comfort improving measures were positively associated with women’s satisfaction.

### Satisfaction with midwife-led prenatal counseling & postpartum guidance

Providing health education, information and emotional support to women are the main components of maternal care [17], and these are what the midwives offer in prenatal counseling and postpartum guidance. Midwife-led prenatal counseling is an important part of prenatal care. Studies have shown that this approach can improve women’s confidence for natural birth. Women tend to consider midwives to be the designated health caregivers for providing prenatal health education [28, 29]. A qualitative study in Sweden found that women experienced a greater sense of calm and preparedness from midwife-led prenatal counseling, and increased their tolerance for the uncertainty related to the birthing process [30]. In our study, counseling was offered by skilled midwives who had at least 10 y of maternity care experience. The content of counseling included rational diet with balanced nutrition, supplements, proper exercise, and recognition of signs of labor. Verbal and written health information is provided. It is shown that women who attend midwife-led prenatal counseling tend to have higher satisfaction score than those who did not, but in our study only 53.6% of women attended the counseling. Obviously, more work needs to be done to increase the number of women attending these counseling sessions.

As for postpartum guidance, our study showed that Midwives’ postpartum guidance is significantly related to women’s satisfaction with childbirth. But there is a problem that must be recognized, unlike other optional care components, such as a doula companion, providing postpartum guidance to all women is a standard of care. In this study, 2.2% of women didn’t think they get postpartum guidance from the midwife. This may be due to the insufficient quality of care, and more high-quality postpartum guidance should be implemented.

### Satisfaction with labor companion

Doula and family member presence at delivery significantly impacts women’s satisfaction of the delivery process. Studies found that labor companionship during childbirth can improve women’s childbirth experience and even birth outcomes [31, 32]. In China, labor companion (partner, family member or doula) is recommended in hospitals by the government policy. This standard is implemented in all first level maternity hospitals. However, institutional practices may not be practical in many other hospitals due to safety concerns. Other barriers to implementing this standard are the insufficient recognition of the benefits of companionship and lack of space or privacy concerns [31]. In our study setting, a maternity care center in Shanghai was able to achieve a remarkable implementation level of 87.7% of labor companionship.

### Pain relief & comfort improving measures versus satisfaction

Labor pain is a complex multidimensional experience and is affected by pain beliefs, tolerance and pain understanding [33]. In our study, pain relief & comfort improving measures such as warm perineal compresses, Lamaze breathing techniques, and epidural anesthesia correlated highly with women’s satisfaction of labor.

Warm perineal compresses used during the second stage of labor are an effective way to reduce pain and third- and fourth- degree perineal lacerations [34, 35]. Our warm compresses are prepared using red bean bags that are sterilized and kept in an incubator at 50 degrees Celsius. The natural properties of red beans, recorded in the Compendium of Materia Medica in China, make them excellent warm perineal compresses. The red bean is beneficial to reduce swelling, is safe and non-toxic.

And the warm compress with red bean has strong penetrating power to tissues, which makes it can enhance blood circulation, improve local metabolism, and promote inflammation dissipating, thus to reduce perineal edema and perineal pain.

The Lamaze breathing techniques were invented by Soviet scientists in the late 1940s [36]. These techniques have become well known worldwide and were originally used for painless childbirth. However, it often fails to achieve painless childbirth. Vojta (one of original of Lamaze method) redefined Lamaze methods’ goals: instead of ensuring complete painlessness in birth, Lamaze now aims to prepare women physically and psychologically for childbirth [36]. In our cohort of patients, we found that the use of Lamaze method positively related to women’s satisfaction with childbirth.

Epidural anesthesia is the gold standard of labor pain relief, and it is specifically effective during the second and third stage of labor [37]. In our hospital epidural anesthesia is administered via an indwelling catheter infusing continuous mixture of low dose local anesthetics and an opioid (0.1% ropivacaine and 0.3% sufentanil). We monitor epidural anesthesia continuously because of its possible risks of maternal hypotension, urinary retention, fever, prolongation of second stage thus total duration of labor [34, 38]. The use of epidural anesthesia is not universal around the World. In India for example the knowledge, attitude, and practice regarding epidural anesthesia among parturient women are different [39]. Kamakshi found that about 33% of the participants knew that delivery is possible without pain, but only 18% were satisfied with the procedure. In Turkey, epidural anesthesia rate for women who have vaginal deliveries is 3.3% [40]. While in the US, the rate is 60.7–83.5% [41]. Our hospital rate as reported here is 75.6% for vaginal births, which is quite similar to that described in the US.

### Episiotomy, perineal laceration

Episiotomy, even though it is a minor incision, is still a surgical incision that can lead to more severe perineal trauma, need for sutures, and possible healing complications [34, 42]. These complications increase pain and decrease women’s satisfaction. In this study, the use of episiotomy was well controlled (23.2%), and the 3rd degree perineal laceration was very insignificant (2 cases out of 4192). This episiotomy rate is well below other countries, such as Turkey (54.1%) [40], Nepal (48.8%) [43], Austria (30.0–39.7%) [44]. It is interesting to note that our findings show no correlation between episiotomy or perineal laceration and labor satisfaction. It is possible that the use of nerve block prior to the episiotomy might have dulled the negative feelings for the procedure. Another possible explanation is the meticulous care and support given by the delivering midwife to those who have episiotomy or perineum laceration. The prenatal and natal care parturient women received has more impact on their satisfaction than the physical events of labor.

### Skin to skin contact

Although our study did not find the relation between mother-infant skin to skin contact with women’s satisfaction. The skin to skin contact with mother after delivery is another standard of care and should not be ignored [34, 45]. The implementation of this standard in our study was far from optimal. Only 40.9% of women with natural vaginal delivery had early skin to skin contact with their babies post-delivery. Our study did not elucidate the reason for this low implementation rate. We can only speculate about the barriers to skin to skin contact. Such barriers might include insufficient staffing, staff time constraint, and safety concerns [45]. Interventions to improve skin to skin contact through training, designating health personnel to help with skin to skin contact and teamwork might improve our compliance rates [45].

Overall, to improve the health care service, the role and competencies of midwives should be utilized. Change in midwife practice, such as expanding the role of midwives, providing maternal care beyond the labor room, such as prenatal counseling and postpartum guidance should be made. Midwifery practice aiming at improving women’s experience during childbirth, such as labor companion, pain relief & comfort improving measures should be recommended. The findings of this study could be important evidence as to expand and improve the health care services where the role and competencies of midwives should be utilized. Since Midwifery-led interventions are associated with efficient use of resources and a positive impact on maternal health outcomes [12, 14] More efforts should be put in policy and system level, to strengthen the capacity of midwives, to improve the midwifery practice and to contribute to the building of sustainable, cost-effective, and equitable health care service [14].

### Limitation

There are several limitations to our study: First, women’s satisfaction with childbirth is subjective and complex. In this study, satisfaction was assessed by a brief and simple item rather than a well-organized questionnaire. Also, women’s experiences were not assessed by a standardized instrument. These might bring bias in the interpretation of the results. A scale with good reliability and validity to measure women’s experience and satisfaction is needed to be developed for future research. Secondly, only Chinese women in a major city (Shanghai) were surveyed. On the one hand, as one of the most economically developed regions in China, the medical resources, practice and concepts in Shanghai differ from many rural areas of China, therefore the results should be generalized with caution. However, on the other hand, Shanghai also has a large amount of transient and floating population. In this study, only 31.3% of the participant are Shanghainese (with local identification). This may increase the representativeness of the sample to a certain extent. Thirdly, some important confounding factors which may affect women satisfaction with childbirth such as length of stay in the hospital were not collected in this study, and this need to be further confirmed by later studies. Moreover, different models of care hadn’t been compared in this study.

## Conclusions

In this study, we investigated the experience and satisfaction of 4192 women who’ve received midwife-led maternity care. With regards to the quality of care, Doula and family member presence during delivery as well as free positioning during the first stage of labor have high emphasis in midwife-led prenatal care (with rates ≥80%). Rate of episiotomy is well controlled (rate 23.2%). However the mother-infant skin to skin contact is far from optimal (rate 40.9%), as well as Midwife-led prenatal counseling (rate 53.6%) which is an important component of midwifery care beyond the labor room. In terms of satisfaction, women generally felt satisfied with midwife-led maternity care. The midwife-led team utilized prenatal counseling, presence of doula and family member, Lamaze breathing techniques, warm perineal compresses, epidural anesthesia, free positioning during the first stage of labor, procedures to promote less pain, and more comfort and postpartum guidance leading to high satisfaction rates for midwife-led maternity care. This study contributed knowledge for understanding the experiences and perspective among birthgiving women in China and the results may be used for improvement in the quality of delivery care as to respond adequately to the women’s needs and to find the most cost-effective care model. And increased coverage of midwife-led maternity care are recommended in health care systems. To achieve this, greater efforts should be placed on midwifery education, training, regulation, and enabling environment.

## Supplementary Information

**Additional file 1.** Experience and Satisfaction with Midwife-led Maternity Care Questionnaire

## Data Availability

The datasets used for analysis during the current study are available from the corresponding author on reasonable request.

## Abbreviations

ANOVA: Analysis of Variance
C-S: Cesarean Section
ICM: International Confederation of Midwives
SPSS: Statistic Package for Social Science
US: United States

## References

[1] Ferrazzi E, Visconti E, Paganelli AM, Campi CM, Lazzeri C, Cirillo F, Livio S, Piola C (2015). The outcome of midwife-led labor in low-risk women within an obstetric referral unit. J Matern-Fetal Neonat Med.

[2] Gu C, Zhu X, Ding Y, Setterberg S, Wang X, Tao H, Zhang Y (2018). A qualitative study of nulliparous women's decision making on mode of delivery under China's two-child policy. Midwifery.

[3] Zhang Z, Gu C, Zhu X, Ding Y, Simone S, Wang X, Tao H (2018). Factors associated with Chinese nulliparous women's choices of mode of delivery: a longitudinal study. Midwifery.

[4] Darsareh F, Aghamolaei T, Rajaei M, Madani A (2018). Exploring first-time pregnant Women's motivations for planning vaginal delivery: a qualitative study. Iran J Nurs Midwifery Res.

[5] Bossano CM, Townsend KM, Walton AC, Blomquist JL, Handa VL: The maternal childbirth experience more than a decade after delivery. Am J Obstet Gynecol 2017, 217(3):342.e341–342.e348. 10.1016/j.ajog.2017.04.027 28455080

[6] Wang E (2017). Requests for cesarean deliveries: the politics of labor pain and pain relief in Shanghai, China. Soc Sci Med.

[7] Liang J, Mu Y, Li X, Tang W, Wang Y, Liu Z, Huang X, Scherpbier RW, Guo S, Li M (2018). Relaxation of the one child policy and trends in caesarean section rates and birth outcomes in China between 2012 and 2016: observational study of nearly seven million health facility births. BMJ.

[8] Wang E, Hesketh T (2017). Large reductions in cesarean delivery rates in China: a qualitative study on delivery decision-making in the era of the two-child policy. BMC Pregnancy Child.

[9] Larsson B, Karlstrom A, Rubertsson C, Ternstrom E, Ekdahl J, Segebladh B, Hildingsson I (2017). Birth preference in women undergoing treatment for childbirth fear: a randomised controlled trial. Women Birth.

[10] Sandall J, Soltani H, Gates S, Shennan A, Devane D (2016). Midwife-led continuity models versus other models of care for childbearing women. Cochrane Database Syst Rev.

[11] de Jonge A, Sandall J (2016). Improving research into models of maternity care to inform decision making. PLoS Med.

[12] Renfrew MJ, McFadden A, Bastos MH, Campbell J, Channon AA, Cheung NF, Silva DR, Downe S, Kennedy HP, Malata A (2014). Midwifery and quality care: findings from a new evidence-informed framework for maternal and newborn care. Lancet.

[13] Hua J, Zhu L, Du L, Li Y, Wu Z, Wo D, Du W (2018). Effects of midwife-led maternity services on postpartum wellbeing and clinical outcomes in primiparous women under China's one-child policy. BMC Pregnancy Child.

[14] Nove A, Friberg IK, de Bernis L, McConville F, Moran AC, Najjemba M, Ten Hoope-Bender P, Tracy S, Homer CSE (2021). Potential impact of midwives in preventing and reducing maternal and neonatal mortality and stillbirths: a lives saved tool modelling study. Lancet Glob Health.

[15] Jiang X-M, Chen Q-Y, Guo S-B, Jin L-Z, Huang X-X, Liu X-W, Hong J-X, Qu H-B, Hu R-F (2018). Effect of midwife-led care on birth outcomes of primiparas. Int J Nurs Pract.

[16] Zhang J, Haycock-Stuart E, Mander R, Hamilton L (2015). Navigating the self in maternity care: how Chinese midwives work on their professional identity in hospital setting. Midwifery.

[17] Zhu X, Yao J, Lu J, Pang R, Lu H (2018). Midwifery policy in contemporary and modern China: from the past to the future. Midwifery.

[18] Huang J, Lu H, Li J, Zhou N, Zang Y, Ren L, Wang J. Comparison of midwives' self-perceived essential competencies between low and high maternal mortality ratio provinces in China. J Clin Nurs. 2020. 10.1111/jocn.15514 32979861

[19] Cheung NF (2009). Chinese midwifery: the history and modernity. Midwifery.

[20] Gao LL, Lu H, Leap N, Homer C (2019). A review of midwifery in mainland China: contemporary developments within historical, economic and sociopolitical contexts. Women Birth.

[21] Homer CS (2016). Models of maternity care: evidence for midwifery continuity of care. Med J Aust.

[22] Gu C, Zhang Z, Ding Y (2011). Chinese midwives' experience of providing continuity of care to labouring women. Midwifery.

[23] Cheung NF, Mander R, Wang X, Fu W, Zhou H, Zhang L (2011). Views of Chinese women and health professionals about midwife-led care in China. Midwifery.

[24] Zeng Y, Hesketh T (2016). The effects of China's universal two-child policy. Lancet.

[25] Li HT, Luo S, Trasande L, Hellerstein S, Kang C, Li JX, Zhang Y, Liu JM, Blustein J (2017). Geographic variations and temporal trends in cesarean delivery rates in China, 2008-2014. JAMA.

[26] Liu X, Lynch CD, Cheng WW, Landon MB (2016). Lowering the high rate of caesarean delivery in China: an experience from Shanghai. BJOG.

[27] Ghasemi A, Zahediasl S (2012). Normality tests for statistical analysis: a guide for non-statisticians. Int J Endocrinol Metab.

[28] Baron R, Heesterbeek Q, Mannien J, Hutton EK, Brug J, Westerman MJ (2017). Exploring health education with midwives, as perceived by pregnant women in primary care: a qualitative study in the Netherlands. Midwifery.

[29] Baron R, Martin L, Gitsels-van der Wal JT, Noordman J, Heymans MW, Spelten ER, Brug J, Hutton EK (2017). Health behaviour information provided to clients during midwife-led prenatal booking visits: findings from video analyses. Midwifery.

[30] Larsson B, Hildingsson I, Ternstrom E, Rubertsson C, Karlstrom A (2019). Women's experience of midwife-led counselling and its influence on childbirth fear: a qualitative study. Women Birth.

[31] Bohren MA, Berger BO, Munthe-Kaas H, Tuncalp O (2019). Perceptions and experiences of labour companionship: a qualitative evidence synthesis. Cochrane Database Syst Rev.

[32] Afulani P, Kusi C, Kirumbi L, Walker D (2018). Companionship during facility-based childbirth: results from a mixed-methods study with recently delivered women and providers in Kenya. BMC Pregnancy Child.

[33] Jones LE, Whitburn LY, Davey MA, Small R (2015). Assessment of pain associated with childbirth: Women's perspectives, preferences and solutions. Midwifery.

[34] Dresang LT, Yonke N (2015). Management of Spontaneous Vaginal Delivery. Am Fam Physician.

[35] Tsakiridis I, Mamopoulos A, Athanasiadis A, Dagklis T (2018). Obstetric anal sphincter injuries at vaginal delivery: a review of recently published National Guidelines. Obstet Gynecol Surv.

[36] Hresanova E (2016). The Psychoprophylactic method of painless childbirth in socialist Czechoslovakia: from state propaganda to activism of enthusiasts. Med Hist.

[37] Czech I, Fuchs P, Fuchs A, Lorek M, Tobolska-Lorek D, Drosdzol-Cop A, Sikora J (2018). Pharmacological and Non-Pharmacological Methods of Labour Pain Relief-Establishment of Effectiveness and Comparison. Int J Environ Res Public Health.

[38] Yin H, Hu R (2019). A cohort study of the impact of epidural analgesia on maternal and neonatal outcomes. J Obstet Gynaecol Res.

[39] Kamakshi G, Anju G, Tania S, Priyanka G, Kamya B, Gegal P, Priyanka C (2018). Epidural analgesia during labor: attitudes among expectant mothers and their care providers. Anesth Essays Res.

[40] Karacam Z, Arslan Kurnaz D, Gunes G (2017). Evaluating the content and quality of intrapartum care in vaginal births: an example of a state hospital. Turk J Obstet Gynecol.

[41] Altman MR, Murphy SM, Fitzgerald CE, Andersen HF, Daratha KB (2017). The cost of nurse-midwifery care: use of interventions, resources, and associated costs in the hospital setting. Women's Health Issues.

[42] Muhleman MA, Aly I, Walters A, Topale N, Tubbs RS, Loukas M (2017). To cut or not to cut, that is the question: A review of the anatomy, the technique, risks, and benefits of an episiotomy. Clin Anat (New York, NY).

[43] Cederfeldt J, Carlsson J, Begley C, Berg M (2016). Quality of intra-partum care at a university hospital in Nepal: a prospective cross-sectional survey. Sex Reprod Healthc.

[44] Marschalek ML, Worda C, Kuessel L, Koelbl H, Oberaigner W, Leitner H, Marschalek J, Husslein H (2018). Risk and protective factors for obstetric anal sphincter injuries: a retrospective nationwide study. Birth.

[45] Alenchery AJ, Thoppil J, Britto CD, de Onis JV, Fernandez L, Suman Rao PN (2018). Barriers and enablers to skin-to-skin contact at birth in healthy neonates - a qualitative study. BMC Pediatr.

