# Supplementary material for: Women’s experience and satisfaction with midwife-led maternity care: a cross-sectional survey in China
Source: BMC Pregnancy Childbirth. 2021 Feb 19;21:151. doi: 10.1186/s12884-021-03638-3 (PMC7893951; doi:10.1186/s12884-021-03638-3)
Supplement: Supplementary file 1 — Additional file 1. Experience and Satisfaction with Midwife-led Maternity Care Questionnaire [file 12884_2021_3638_MOESM1_ESM.docx]

**Experience and Satisfaction with Midwife-led Maternity Care Questionnaire**

**Section 1: General Information**

1.Your age: years old.

2. Your household registration location: ⬜ Shanghai ⬜ Non-Shanghai

3. Is this your first time giving a birth? ⬜Yes ⬜ No

4. Your gestational age: weeks days

5. Gender of your newborn: ⬜ Boy ⬜ Girl; Weight of your newborn: g.

**Section 2 Experience**

1. Did you receive the midwife-led prenatal counseling?

⬜ Yes ⬜ No

2. Did you have a doula or a family member present at delivery?

⬜ Yes ⬜ No

3. Did you use Lamaze breathing techniques to reduce pain during labor?

⬜ Yes ⬜ No

4. Did you get warm perineal compress with red-bean bag before delivery?

⬜ Yes ⬜ No

5. Did you use Epidural anesthesia during labor?

⬜ Yes ⬜ No

6. Did you use free position during the first stage of labor?

⬜ Yes ⬜ No

7. Did you use free position during the second stage of labor?

⬜ Yes ⬜ No

8. Was lateral episiotomy performed for you during delivery?

⬜ Yes ⬜ No

9. Did the midwife perform mother-infant skin to skin contact for you and your baby right after delivery?

⬜ Yes ⬜ No

10. Did you have a perineal laceration?

⬜ No

⬜ Yes, and I had a lst degree laceration

⬜ Yes, and I had a 2nd degree laceration

⬜ Yes, and I had a 3rd degree laceration

11. How do you feel about the pain in the perineum?

⬜ Almost no pain

⬜ Mild pain

⬜ Moderate pain

⬜ Severe pain

12. Do you have any edema in your perineum?

⬜ Almost no edema

⬜ Mild edema

⬜ Moderate edema

⬜ Severe edema

13. Did you get postpartum guidance from midwives?

⬜ Yes ⬜ No

**Section 3 Satisfaction**

Please choose a number from 1 to 10 to describe your overall satisfaction with the childbirth experience(1 refers to not satisfied, and 10 refers to completely satisfied).

| 1 | 2 | 3 | 4 | 5 | 6 | 7 | 8 | 9 | 10 |
| --- | --- | --- | --- | --- | --- | --- | --- | --- | --- |
|  |  |  |  |  |  |  |  |  |  |
